# Supplementary material for: Associations among weed communities, management practices, and environmental factors in U.S. snap bean (Phaseolus vulgaris) production
Source: PLoS One. 2025 Sep 23;20(9):e0332925. doi: 10.1371/journal.pone.0332925 (PMC12456788; doi:10.1371/journal.pone.0332925)
Supplement: Attachment — Submitted filename: Response to Reviewers.docx [file pone.0332925.s002.docx]

**Response to Requirements and Reviewers for Manuscripts PONE-D-25-08867**

**NOTE:** The line numbers in my comments (in red) are following the line numbering of the revised manuscript without tracking changes. The line numbering of the comments from reviewers follows the line numbering pattern of the original manuscript submission.

Journal Requirements:

1. When submitting your revision, we need you to address these additional requirements.

Please ensure that your manuscript meets PLOS ONE's style requirements, including those for file naming. The PLOS ONE style templates can be found at

<https://journals.plos.org/plosone/s/file?id=wjVg/PLOSOne_formatting_sample_main_body.pdf> and

[https://journals.plos.org/plosone/s/file?id=ba62/PLOSOne_formatting_sample_title_authors_affiliations.pdf](https://track.editorialmanager.com/CL0/https:%2F%2Fjournals.plos.org%2Fplosone%2Fs%2Ffile%3Fid=ba62%2FPLOSOne_formatting_sample_title_authors_affiliations.pdf/1/010f019660760675-d69d277f-f5e1-4a0b-b229-920f36277e76-000000/e08_8xk5lsEYtZM3reOWqyIDktccOZ05iMSYDaEoUrI=208)

The necessary fixes regarding formatting of each section, abstract and author list have been made according to the guidelines provided.

2. In your Methods section, please provide additional information regarding the permits you obtained for the work. Please ensure you have included the full name of the authority that approved the field site access and, if no permits were required, a brief statement explaining why.

Explanation provided in the Materials in Methods section in L94-97.

3. Please note that PLOS ONE has specific guidelines on code sharing for submissions in which author-generated code underpins the findings in the manuscript. In these cases, we expect all author-generated code to be made available without restrictions upon publication of the work. Please review our guidelines at https://journals.plos.org/plosone/s/materials-and-software-sharing#loc-sharing-code and ensure that your code is shared in a way that follows best practice and facilitates reproducibility and reuse.

I have analyzed all my data using R with RStudio as IDE (R version 4.4.0, corrected in manuscript in L148, and RStudio version 2025.05.1+513). I have the author-generated code that I used for data curation and analysis. R is free software, so I am more than happy to share the code file if that is a requirement of reviewers, please let me know. Since, I do not fully understand the repository software sharing rules. I would like further clarification on this point.

4. Thank you for stating the following financial disclosure: [This research was funded by U.S. Department of Agriculture–Agricultural Research Service project 5012-12220-010-000D (“Resilience of Integrated Weed Management Systems to Climate Variability in Midwest Crop Production Systems”). Any opinions, findings, conclusions, or recommendations expressed in this publication are those of the authors and do not necessarily reflect the views of the U.S. Department of Agriculture. The mention of trade names or commercial products in this publication is solely for the purpose of providing specific information and does not imply recommendation or endorsement. The U.S. Department of Agriculture is an equal opportunity provider and employer.].

Please state what role the funders took in the study.  If the funders had no role, please state: ""The funders had no role in study design, data collection and analysis, decision to publish, or preparation of the manuscript.""

If this statement is not correct you must amend it as needed.

Please include this amended Role of Funder statement in your cover letter; we will change the online submission form on your behalf.

Statement "The funders had no role in study design, data collection and analysis, decision to publish, or preparation of the manuscript." Included in the amended Role of Funder statement, now included in the amended cover letter file Cover_Letter_PP_Amended

5. We note that you have included the phrase “data not shown” in your manuscript. Unfortunately, this does not meet our data sharing requirements. PLOS does not permit references to inaccessible data. We require that authors provide all relevant data within the paper, Supporting Information files, or in an acceptable, public repository. Please add a citation to support this phrase or upload the data that corresponds with these findings to a stable repository (such as Figshare or Dryad) and provide and URLs, DOIs, or accession numbers that may be used to access these data. Or, if the data are not a core part of the research being presented in your study, we ask that you remove the phrase that refers to these data.

“data not shown” removed in L227

“data not shown” in L262 changed to reference [19] as that manuscript presents and visualizes weed densities.

6. Please include captions for your Supporting Information files at the end of your manuscript, and update any in-text citations to match accordingly. Please see our Supporting Information guidelines for more information: <http://journals.plos.org/plosone/s/supporting-information>.

Captions for the .csv file containing data used for analysis added in the Supporting Information section at the end of the manuscript.

7. Please review your reference list to ensure that it is complete and correct. If you have cited papers that have been retracted, please include the rationale for doing so in the manuscript text, or remove these references and replace them with relevant current references. Any changes to the reference list should be mentioned in the rebuttal letter that accompanies your revised manuscript. If you need to cite a retracted article, indicate the article’s retracted status in the References list and also include a citation and full reference for the retraction notice.

I have ensured that the reference list is complete and correct. References [24] and [25] have switched places, as the inclusion of an additional sentence moved reference [25] ahead of the past reference [24].

**Reviewer #1**

This manuscript presents a valuable investigation into the complex interplay between weed communities, management practices, and environmental factors in US snap bean production. The use of machine learning (random forest) to analyze large-scale field survey data represents a robust methodological approach, and the findings offer practical insights for optimizing weed control and yield outcomes. However, several critical areas require improvement to strengthen the study’s validity, interpretability, and alignment with journal standards:

1. While random forest excels in capturing nonlinear relationships, compare its performance against simpler statistical models (e.g., linear regression, GLM) to contextualize its advantages. Present metrics such as R², RMSE, or AIC to justify the choice of machine learning.

2. Acknowledge that your findings are based on mechanized, high-input systems in three US regions. Discuss the limitations to scalability, particularly for smallholder or organic systems where tillage practices, herbicide use, and climate stressors differ. Highlight opportunities to adapt these insights to diverse contexts (e.g., integrating machine learning with low-tech monitoring tools in resource-limited settings).

1. We justify the application of this machine learning method as you stated before because of its robustness. We have included references to some previous papers that have at the very least done a very similar broad-scale look at agronomic production of certain crops and have shown that random forest was the most parsimonious approach. The newly included reference sentence can be found in L142-143 of the manuscript. The reason why we used this approach is due to having numerous predictor variables that belong to different data types, making many of the underlying relationships non-linear by default. Also, due to its non-parametric nature it isn’t really possible to compare metrics to those of parametric approaches, perhaps to other non-parametric approaches it would make sense, but even the underlying math could be entirely different. We fail to understand how additional analysis will add new insight to the data as random forest isn’t exactly a novel approach. It is novel in this type of large scale survey in snap bean production, especially in relation to weed science. We are simply building upon the past weed survey work where decision trees were used and have opted to use a more powerful algorithm.

2. We have clearly stated from the beginning that our manuscript and collected data comes from commercial high-input systems. Specifically, it is only snap bean production that is used for processing purposes which we have also clearly delineated from fresh market purposes, as no such fields were used in the survey. Commercial production may not represent organic systems where herbicides are not available, therefore we believe that we cannot really comment on organic production systems. We didn’t want to mix these two production systems, so there are no further comments regarding scalability or other limitations.

Line 126-131: Why aggregate precipitation into 3 intervals? Justify this choice statistically/methodologically.

Methodologically our reasoning is to have a somewhat finer approach to three general periods of crop growth and development being covered. This is very useful as the crop itself has a very short growing season, making it so that they have more of a substantial effect on the crop. We are generalizing crop growth with this approach as we don’t have a finer picture of how the crop looked like in these stages for each field.

Line 132: "machine learning algorithm random forest". Specify "random forest regression" for clarity, as machine learning encompasses diverse methods.

Fixed.

Line 284-293: You attribute NW’s higher yield to "interaction of factors." Elaborate on these factors (e.g., soil moisture, pest pressure).

Irrigation, well drained soils, low disease incident etc. added in L300. This has been further elaborated in Results and discussion previously.

Line 299-300: The conclusion overstates climate impacts without projecting future scenarios. Recommend adding a brief discussion on how your findings inform adaptive strategies.

Could you further elaborate on this point based on how I am re-reading the conclusion we aren’t necessarily overstating the impact of climate. The goal of this paper wasn’t to discuss the impact of climate or climate change and make future projections regarding its effect on snap bean production. We just found through our analysis that temperature plays an important role in predicting snap bean yield, which is a realistic expectation irrespective of the crop. Hence the reason why we state that there aren’t many adaptive strategies that growers can necessarily adopt. We believe that projecting the effects of climate change on snap bean production should warrant further investigation, but do not claim that as of right now we have the information what the best strategy would be. We are simply stating what the best strategy would be based on the currently well-established practices of growing snap bean and weed management.

Fig 2A. The Northwest (NW) outperforms the Midwest (MW). Could soil type (e.g., sandy vs. clay) or climate adaptation (e.g., heat tolerance) explain this difference?

Could you elaborate further on this? As I am re-reading the paragraph, we do state how many of these different factors are potentially affecting the yield differences we are seeing. We use both the data from actual surveys to demonstrate that (soil texture differences) and also reference past works conducted in snap bean to explain why we are most probably seeing these differences. We are taking this approach as the work conducted on snap bean isn’t as extensive as in some of the other crops, so we are basing our conjectures on what we know and understand so far about the crop and based on what we know when it comes to any kind of crop production as similar lines can be drawn. Lastly, we don’t necessarily claim to have all the answers based on the data we have collected and presented, as this is the first and only kind of large-scale outlook on snap bean production ever conducted. So further inquiry into some of the results we are seeing would and should warrant more high resolution investigation.

**Reviewer #2**

This manuscript presents an analysis on the relationships between management and environmental factors of snap bean fields with crop yield and weed density across the three production regions in the United States collected from survey data using random forest modelling. The authors found region to be the most important predictor of crop yield followed by planting date and whether the producer used row cultivation. Row cultivation and the number of spring tillage operations were the most important predictors of weed density. The introduction does not present a compelling story and could use some reworking. First, it would be beneficial to mention the problem this study addresses earlier rather than in the final paragraph. At first it seems this manuscript will be about shifts in management practices by snap bean producers as the first paragraph does not even mention weeds (or any pest) and most details concern weed impact on bean yields in general and effects of climate change on snap bean and weed management. I think there needs to be greater emphasis on the why here. This is not the first study to link environmental and management practices to weed density and crop yield; have others been successful? Further, why did the authors choose a ML random forest approach? Has this been used to model weed density and crop yield in the past? The methodology and analysis is appropriate for the research question yet no information is provided on why this particular approach was selected. I.e. why random forest modeling and why not another decision tree modeling approach? The results support the conclusions and will be of interest to a wide range of readers. With several small additions I believe this manuscript will be a valuable contribution to PLOS.

Thank you for your kind words about the value of our work. I think the concept of our work has more to do with using the data we have collected to produce results and present the story in regard to that. Our work isn’t necessarily stating a specific hypothesis or problem but rather is presenting large-scale picture regarding snap bean production in the United States, which makes this an original work first of its kind which we would like to be useful and beneficial to future researchers. Especially if they would like to explore certain aspects in a higher resolution. We haven’t found many studies that link these factors in a similar way we do, but we have included some references in the Materials and methods section and provided a case why random forest was the algorithm of choice.

L92-94 – How were producers and fields selected for this survey? If it was vegetable processors who were contacted would there be a worry about potential bias?

Snap beans are mostly grown under contract by vegetable processors. Therefore, it is necessary to go through processors in order to obtain the location of fields. The processors provided us with multiple fields, and we weren’t always able to visit all the fields, sometimes having to randomly choose which ones we will survey. It is true that our choices weren’t wide, but this is inherent to the scale of snap bean production in the surveyed states. This is a specialty crop, hence the areas it is grown on aren’t large, so any field that we could collect the data from we did so. Overall, there was no specific criteria in which fields were surveyed, except that they are snap beans, under conventional (non-organic) production system and used for processing purposes.

L100 – If fields were surveyed near harvest time and surveys happened from June to Oct does this mean snap beans are continuously harvested for 5 months? Did the authors survey different regions during different times? I see confounding issues here with sampling over what is essentially the entire growing season.

Snap beans are planted at different times in different regions and sometimes are planted a second time in the middle of the summer. We stated in our model analysis that we do see a positive effect of later planting dates on higher across regions, as partial dependency plots do marginalize across other predictors. However, we did state that somewhat later planting dates in NW regions probably have an effect on higher yields there. We also do state that it is probably a combination of multiple different factors not just the planting date.

L104-106 – How were quadrats placed in fields? Were they equally spaced? How did the authors ensure they were capturing variability across the field?

We have actually provided a reference [19] in L112 to our previously published work and how the data was collected in those surveys regarding weed populations.

L105 – What is a species group? How did the authors decided which to pool and which not too?

We have actually provided a reference [19] in L112 to our previously published work and how the data was collected in those surveys regarding weed populations.

L124-125 – What physical and chemical properties?

Explained further in L130-132.

L143 – How was weed cover % determined?

Explained in L111-112 with reference [19].

L152 – Scaled how?

Clarification provided in L162-163.

L153 – Scaled and transformed how?

Clarification provided in L162-163.

L175-178– I think this is important background material missing from the introduction.

I decided to leave this information at the current manuscript section as it ties in more with the discussion regarding the determination of crop yield.

L183-186 – What about the authors data?

Reference to [20] added in L197 regarding author-obtained data.

L198 – On weed populations?

Fixed.

L234 – I think it is obvious different cultivation equipment can cause varying levels of crop damage.

Could you elaborate further on this point, since I fail to see what would be the necessary revision?

L248 – Is that decline really only 1 plant/m2? It looks closer to 2 but this figure is not easy to interpret. Would a decline in weed density of 1 plant/m2 for row cultivation make it worth it? This seems very low to me.

The decline is closer to ~3 plants/m^2^. The figures have been adjusted for clarity and readability.

L297 – Is a 1 plant/m2 decline in weed density a beneficial practice when this is accompanied by yield loss?

As stated previously the decline is closer to ~3 plants/m^2^. This may warrant further investigation when it comes to this interesting trade-off we have observed. However, what we can state is that this decrease in weed density is important. Weeds can be very competitive, so even if row cultivation may come at the cost of a slight yield loss to the crop, it could still prove beneficial in the long run due to the substantial loss in yield due to the said competition.

All figures are all very low resolution. Please fix.

Figure letter captions are inconsistent, some in top left others in top right, others missing entirely (Fig 3)

Fig 2A, Fig 2C and Fig 4A - are these correct? They only two dots each. Perhaps a box and whisker plot would be better to show distribution of yield and weed density by region and with and without cultivation.

Fig 3 – What is the y-axis on these?

What is the y-axis on Fig 4B? Is it weed density as well? Is this a regression line or just connecting non-continuous points? Further, there is no measure of variability presented.

Figure resolution has been fixed; hence they should be clearer and more readable. Caption inconsistency has been fixed in all figures and more details have been provided for better clarity of figure elements. The lines (or dots) that have been drawn on Fig2 and Fig4 are partial dependency plots. Since they marginalize over the other predictors, they give a function that only depends on the chosen predictor from the trained random forest model. This has been explained in Materials and methods section (L170-173). Therefore, they are not regression lines or box and whisker plots and there is no showing of variability in them.
