# Supplementary material for: Associations among weed communities, management practices, and environmental factors in U.S. snap bean (Phaseolus vulgaris) production
Source: PLoS One. 2025 Sep 23;20(9):e0332925. doi: 10.1371/journal.pone.0332925 (PMC12456788; doi:10.1371/journal.pone.0332925)
Supplement: Attachment — Submitted filename: Response to Reviewers (2).docx [file pone.0332925.s003.docx]

**Response to Requirements and Reviewers for Manuscripts PONE-D-25-08867**

**NOTE:** The line numbers in my comments (in red) are following the line numbering of the revised manuscript without tracking changes. The line numbering of the comments from reviewers follows the line numbering pattern of the original manuscript submission.

L105-109 – How were quadrats placed in fields? Were they equally spaced? How did the authors ensure they were capturing variability across the field?

I addressed this comment previously and this is what I originally mentioned:

“We have actually provided a reference [19] in L112 to our previously published work and how the data was collected in those surveys regarding weed populations.”

But if we are just focusing on these lines, I have used the methodology described by Thomas (1985) [reference 21]. Where we stayed away from the edges of the field and aimed at spacing out our quadrats as evenly as we could, but we still accounted for weed distribution of the whole field area as best as we could. As we considered if there was consistent patchiness of some weed species, as we didn’t just go for some random single patch that may have been there in an entirety of the field area. Therefore, our statement in using the methodology from Thomas (1985) but with some slight modifications such as considering the weed distribution of the field. Therefore, we could say that there was some randomization in how we placed the quadrats and collected data, but we did somewhat account for representativeness, so it isn’t full randomization. Meaning there is some inherent bias in data sampling, but it is based on agronomic expertise and experience. We have adjusted the lines for clarification.

L109 – What is a species group? How did the authors decide which to pool and which not too?

Added a reference to our previous work [19] in how we decided for species grouping. Which also goes into more detail in how we did the actual sampling regarding the weed community data.
